# The type of bottleneck matters: Insights into the deleterious variation landscape of small managed populations

**DOI:** 10.1111/eva.12872

**Published:** 2019-09-30

**Authors:** Chiara Bortoluzzi, Mirte Bosse, Martijn F. L. Derks, Richard P. M. A. Crooijmans, Martien A. M. Groenen, Hendrik‐Jan Megens

**Affiliations:** ^1^ Department of Animal Sciences, Animal Breeding and Genomics Wageningen University & Research Gelderland The Netherlands

**Keywords:** deleterious variants, domestication, effective population size, population bottleneck, runs of homozygosity, small population

## Abstract

Predictions about the consequences of a small population size on genetic and deleterious variation are fundamental to population genetics. As small populations are more affected by genetic drift, purifying selection acting against deleterious alleles is predicted to be less efficient, therefore increasing the risk of inbreeding depression. However, the extent to which small populations are subjected to genetic drift depends on the nature and time frame in which the bottleneck occurs. Domesticated species are an excellent model to investigate the consequences of population bottlenecks on genetic and deleterious variation in small populations. This is because their history is dominated by known bottlenecks associated with domestication, breed formation and intense selective breeding. Here, we use whole‐genome sequencing data from 97 chickens representing 39 traditional fancy breeds to directly examine the consequences of two types of bottlenecks for deleterious variation: the severe domestication bottleneck and the recent population decline accompanying breed formation. We find that recently bottlenecked populations have a higher proportion of deleterious variants relative to populations that have been kept at small population sizes since domestication. We also observe that long tracts of homozygous genotypes (runs of homozygosity) are proportionally more enriched in deleterious variants than the rest of the genome. This enrichment is particularly evident in recently bottlenecked populations, suggesting that homozygosity of these variants is likely to occur due to genetic drift and recent inbreeding. Our results indicate that the timing and nature of population bottlenecks can substantially shape the deleterious variation landscape in small populations.

## 1 INTRODUCTION

Deleterious mutations are expected to be held at low frequency by an equilibrium between the rate at which they arise by mutation and the efficacy of purifying selection at removing them from the population (mutation–selection balance) (Lynch, 2010; Ohta, 1992). However, the number and frequency of deleterious genetic variants segregating in a population are affected by many evolutionary forces, including artificial selection, genetic drift and genetic hitchhiking, which is the change in allele frequency of a variant that is passed along together with another variant under positive selection (Charlesworth, 2006; Smith & Haigh, 1974).

In small populations, the mutation–selection balance is challenged by population contractions, which reduce the efficacy of purifying selection to remove harmful mutations (Ohta, 1973). As a result, the genetic load, defined as the reduction in mean fitness in a population caused by deleterious variation relative to a mutation‐free population (Kimura, Maruyama, & Crow, 1963), is predicted to be larger. In the long‐term, the high genetic load and the rapid increase in frequency of harmful mutations could impact population survival and genetic diversity, increasing the risk of inbreeding depression (Kimura et al., 1963).

Genetic drift, or the random fluctuations in the number and frequency of alleles, is mostly responsible for the deleterious genetic landscape in small populations. However, as studies in plant and animal species have suggested, the extent to which small populations are subjected to genetic drift considerably varies depending on the nature and time frame in which the bottleneck occurs (Liu, Zhou, Morrell, & Gaut, 2017; Mardsen et al., 2016; Zhang, Zhou, Bawa, Suren, & Holliday, 2016). For instance, a long‐term population decline is expected to result in a lower proportion of amino acid changing variants, along with a reduction in the additive genetic load, due to purifying selection acting against deleterious variants (Mardsen et al., 2016). However, if populations have undergone recent and sudden declines, deleterious variation is predicted to be mainly shaped by genetic drift (Ohta, 1973).

Domesticated species are an excellent model to investigate the consequences of population bottlenecks on genetic and deleterious variation. This is because their demographic history is characterized by multiple population contractions associated with domestication, breed formation and intense selective breeding (Bosse, Megens, Derks, Cara, & Groenen, 2019; Makino et al., 2018; Mardsen et al., 2016; Moyers, Morrell, & McKay, 2017). Domestication involves the (partial or complete) isolation of a number of individuals from a wild progenitor population and entails drastic changes in the nature and strength of selective forces acting on the population, as well as its size (Larson & Fuller, 2014). The domestication bottleneck is usually followed by a long period of relatively weak and varying artificial selection, during which the reduced *N_e_* may either be stable or fluctuate depending on human‐driven selection. Contrary to the long‐term domestication process, breed formation is a more recent event that often entails intense selection over short time periods and is coupled with limited recombination and an additional reduction in *N_e_* (Moyers et al., 2017). In this study, traditional fancy breeds of chicken were used as a model species to investigate the consequences of two types of bottlenecks on deleterious variation: the severe domestication bottleneck occurred some thousands of years ago and the recent population decline accompanying breed formation in the last decades.

Since their development in the 16th and 18th century (Dana et al., 2011), traditional fancy breeds have persisted at small population sizes and comprised normal‐sized (large fowl) and miniature (bantam) breeds. These traditional breeds experienced domestication only, which was based upon preferential breeding of birds exhibiting specific morphological features. The subsequent long period of weak and varying artificial selection resulted in the foundation of numerous breeds that are nowadays identified by an accurate phenotypic description (Tixier‐Boichard, Bed'hom, & Rognon, 2011). In the last decades, hobby breeders have become interested in miniature forms of historical large breeds, which are called neo‐bantams, and were initially created by crossing a large fowl with a bantam individual. Even though mating between neo‐bantams has recently started to become very popular among hobby breeders, the selection purpose of obtaining an individual exhibiting all of the standard large fowl characteristics still remains (Bortoluzzi et al., 2018). The recent creation of neo‐bantam breeds involved, on top of domestication, an additional population bottleneck. As we showed in our previous study, the reduced *N_e_* and parent–offspring mating pursued within a neo‐bantam breed to consolidate favourable traits considerably increased the level of inbreeding (Bortoluzzi et al., 2018). Although we expect the recent and sudden bottleneck to have acted differently on the accumulation of deleterious variants relative to the domestication bottleneck experienced by historical breeds, its effect on genome‐wide patterns of deleterious variation remains unclear.

Accurate predictions of deleterious variants are essential when assessing their contribution to phenotypic variation (Kono et al., 2016). To date, numerous approaches have been developed and applied to nonhuman species (Liu et al., 2017; Makino et al., 2018; Mardsen et al., 2016; Renaut & Rieseberg, 2015; Robinson et al., 2016; Zhang et al., 2016), of which the Sort Intolerat From Tolerant (SIFT) approach is among the most widely used. However, as shown in Kono et al., (2016) and Derks et al. (2018), additional filtering criteria should be applied to the set of deleterious mutations to improve the reliability of the prediction. These criteria should include orthologous genes to minimize the effect of off‐site mapping of sequence reads, RNA expression of protein‐coding variants and the use of different prediction approaches (Derks et al., 2018; Kono et al., 2016). We here expanded the approach of Derks et al. (2018) to predict deleterious mutations in domestic chickens by addressing a potential source of bias not previously investigated. That is reference bias, which is the higher probability of calling a variant as reference. We corrected for that by polarizing all protein‐coding variants by ancestral and derived state, rather than reference and nonreference, to not underestimate the inferred number of nonsynonymous and deleterious variants.

Whole‐genome sequencing data from 97 chickens representing 39 traditional fancy breeds were here used to directly examine the impact of different population bottlenecks on patterns of deleterious variation in small populations. Overall, we find that the recent population bottleneck associated with the creation of neo‐bantams has resulted in a higher proportion of deleterious variants relative to large fowl and bantam counterparts, as genetic drift has reduced the efficacy of purifying selection to eliminate harmful mutations. We also observe that most deleterious variants are found in long tracts of homozygous genotypes, suggesting that homozygosity of these variants is likely to occur due to genetic drift and recent inbreeding. Our results indicate that the time frame and nature of the bottleneck can substantially shape the deleterious variation landscape in small populations.

## 2 MATERIALS AND METHODS

### 2.1 Samples and sequencing

**Figure 1:**
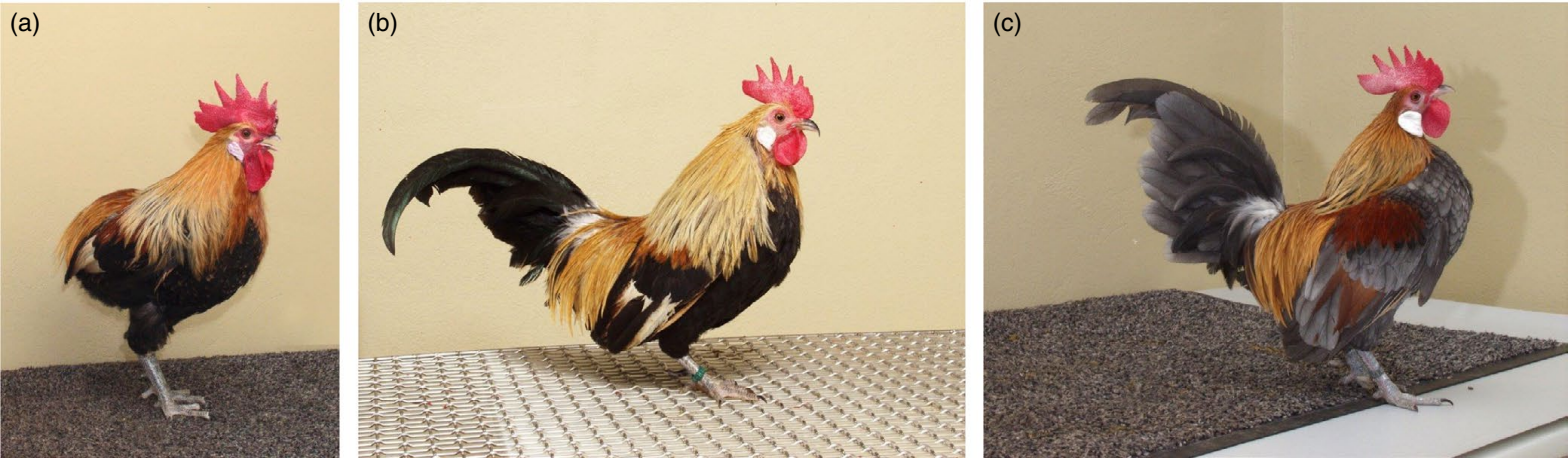
Traditional Dutch chicken breeds. (a) Large fowl. The bird shown here is a Drenthe fowl boolstat, a breed of chicken whose main trait under selection is the absence of the tail. (b) Neo‐bantam. The individual is a bantamised bird of the Dutch fowl breed and thus called Dutch fowl bantam. Neo‐bantams are usually 2/3 the size of the original large fowl counterpart. (c) Bantam. The bird shown is the Dutch bantam, one of the few true bantam breeds that exists only small in size. Bantam chickens are usually about a third to half the size of a regular large fowl chicken

DNA of 97 individuals from 39 traditional chicken breeds from the Netherlands was used for whole‐genome sequencing on an Illumina HiSeq 3,000. Four samples from each of the known living *Gallus* species were also sequenced for this study (Table S1). Based on their demographic and selection history, samples were classified into large fowls (*n* = 51) (Figure 1a), neo‐bantams (*n* = 39) (Figure 1b) and bantams (*n* = 7) (Figure 1c). Sequence reads were processed using standard bioinformatic pipelines (Appendix S3), including alignment to the chicken reference genome (GenBank Accession: GCA_000002315.3; Warren et al., 2017) using the Burrows–Wheeler Aligner (BWA; Li & Durbin, 2010), indel realignment, variant calling and filtering of variants with quality <30. As two samples were discarded from further analyses because of low genome coverage (<5×), the final data set comprised 99 individuals (95 samples from traditional breeds and four samples from the *Gallus* species).

### 2.2 Principal component analysis

Genetic relationships among the 95 sequenced individuals (*Gallus* species excluded) were investigated through a principal component analysis (PCA) performed on the filtered vcf files in PLINK v.1.9 (Purcell et al., 2007). First and second principal components were plotted using R v.3.2.0 (R Core Team, 2013).

### 2.3 Heterozygosity analysis

Individual heterozygosity was estimated for each of the 95 chickens on a genome‐wide scale by dividing the genome into nonoverlapping windows of 10 kb (Table S2 in Appendix S1). Within each window, heterozygous variants were called only if their depth of coverage met the minimum and maximum threshold, which were set at four and two times the average coverage, respectively (Figure S2). The total number of heterozygous sites called in a 10‐kb window was corrected for the total number of sites not called because of low coverage, following (Bosse et al., 2012). Insufficiently covered bins (10‐kb windows with less than 1,000 well‐covered sites) were excluded from the genome‐wide autosomal heterozygosity analysis.

### 2.4 Runs of homozygosity (ROHs)

Runs of homozygosity were extracted from the genome of the 95 sequenced individuals implementing the method developed by (Bosse et al., 2012). Information on heterozygosity was used to identify autosomal ROHs, which are here defined as genomic regions showing lower heterozygosity than expected based on the genome‐wide average. To identify ROHs, we considered 10 consecutive bins at a time (100,000 bps) in which we calculated the average window heterozygosity that was then compared to the average genome‐wide heterozygosity. The 10 consecutive bins were retained as candidate ROHs only if their level of heterozygosity was below 0.25 the average genomic diversity. All 10 consecutive bins that did not meet these criteria were considered to contribute to the genome‐wide heterozygosity level outside ROHs. Local assembly or alignment errors were avoided as much as possible by relaxing the threshold within candidate homozygous stretches allowing for maximally twice the average heterozygosity in a bin, only if the heterozygosity within the candidate ROH did not exceed 1/3 the average genomic diversity (for more information refer to [Bosse et al., 2012]). Insufficiently covered bins were not considered in the actual size of each ROH but were considered in the calculation of the actual ROH length (assuming that all bins were highly covered). ROHs with insufficient coverage (less than 2/3) were removed from our calculations. From the final list of individual ROHs (Table S2 in Appendix S1), we classified ROHs into three size classes, each of them corresponding to a specific demographic event, including past relatedness (short ROHs: <100 kb), background relatedness (medium: 0.1–3 Mb) and recent relatedness (long: ≥3 Mb).

### 2.5 Population history estimation

SMC++ was used on unphased whole‐genome sequencing data to estimate population history (Terhorst, Kamm, & Song, 2017). Only samples with an average genome coverage >10× and percentage of missing sites <10% were considered (Table S5). Population history was estimated for each breed separately setting the mutation rate to 1.9 × 10^‐9^ site^‐1^ year^‐1^ (Nam et al., 2010) and generation time at 1 year.

### 2.6 Inferring the ancestral state

Sequencing data of the three wild *Gallus* species included in the data set (i.e. *G. varius, G. sonneratii and G. lafayetii*) were used as an outgroup to predict the ancestral and derived allelic state of all polymorphic sites. A variant was categorized as ancestral if the three wild samples had the same genotype (homozygous reference or homozygous alternative). The 11,706,316 identified variants were extracted from each sample with a minimum average genome coverage >10× and classified as homozygous ancestral, heterozygous or homozygous derived.

### 2.7 Functional annotation of variants

Variant annotation was performed with the Variant Effect Predictor (VEP) (McLaren et al., 2016) running the Sort Intolerant From Tolerate (SIFT) algorithm, using the Ensembl *Gallus gallus* annotation database (release 90). Protein‐coding variants were defined based on their SIFT score as synonymous, nonsynonymous tolerated (SIFT score ≥0.05), and nonsynonymous deleterious (SIFT score <0.05). We also catalogued mutations that disrupt the generation of a fully functional protein either by introducing a stop codon or by truncating the protein reading frame as loss of function (LoF) variants. To be more confident on the detection of deleterious variants, we implemented the approach developed by (Derks et al., 2018) considering only variants annotated in genes that were 1:1 orthologous in Ensembl with zebra finch and for which the RNA‐seq expression coverage was at least 200 in the Ensembl merged RNA‐seq data set (release 86). To increase our confidence in the deleteriousness of our set of putatively deleterious variants predicted by SIFT, we used the GERP scores computed for the seven sauropsids multiple whole‐genome alignment as an additional approach (ftp://ftp.ensembl.org/pub/release-94/compara/). Variants were considered truly deleterious if SIFT score <0.05 and GERP score >1.0.

### 2.8 Test for elevated homozygosity of derived genotypes

Following (Robinson et al., 2016), we used likelihood ratio tests to evaluate whether the number of homozygous derived genotypes per individual differed between large fowls and neo‐bantams at synonymous, tolerated, deleterious and LoF variant sites (Appendix S3). We focused on these two management groups only as they were previously found to be genetically more similar than to bantam breeds (Bortoluzzi et al., 2018). Therefore, by testing for differences in homozygosity of derived genotypes we wanted to see whether these two groups might also share similar proportion of deleterious variants. Briefly, the test compared the likelihoods under two models. Under the null model, we assumed a similar proportion of homozygous derived alleles between neo‐bantams and large fowls ($p_{\text{lf}}{\;\text{= p}}_{\text{nb}}$), whereas under the alternative model differences in the number of homozygous derived genotypes are expected between large fowls and neo‐bantams ($p_{\text{lf}}\neq p_{\text{nb}}$). The log‐likelihood values of both the null and alternative models were used to calculate the likelihood ratio test (LRT) as, $\Lambda =-2\left(\log{\text{likelihood}}_{\text{null}}-\log{\text{likelihood}}_{\text{alternative}}\right)$.

### 2.9 Genetic load

Genetic load was calculated as the ratio of nonsynonymous deleterious to synonymous sites in each individual and averaged across individuals within each of the three management groups. Genetic load was separately estimated for heterozygous and homozygous derived alleles.

### 2.10 Site‐frequency spectrum (SFS)

The derived allele frequency (DAF) spectrum was calculated for synonymous, tolerated and deleterious variants, considering only bi‐allelic SNPs. We then generated a histogram with 10 bins (with steps of 0.1 allele frequency) starting from a very low (0–0.10) to a very high (0.90–1.0) derived allele frequency.

### 2.11 Enrichment of ROHs for deleterious variants

The distribution of putatively deleterious mutations inside and outside of ROHs was investigated following the method proposed by Szpiech et al. (2013). Homozygous derived variants were grouped into nondamaging or putatively neutral (e.g. synonymous and tolerated) and damaging (e.g. deleterious and LoF). The occurrence of damaging and nondamaging variants was investigated inside and outside each ROH size class. Coordinates of ROHs were used to calculate the fraction of the genome covered by any ROH and by each ROH size class as:

$$G_{\text{i,j}}=\frac{L_{\text{ROH}}}{L_{g}}$$

where $L_{g}$ is the total length of the genome, $L_{\text{ROH}}$ is the total length of ROHs, i is the individual, and *j* is the ROH class jò(S,M,L,A) representing small, medium, long and any ROH, respectively (Table S3 in Appendix S1).

## 3 RESULTS

Patterns of deleterious variation were investigated using whole‐genome sequencing (WGS) of 39 traditional chicken breeds (Table S1). On average, 13.4× coverage was generated for each individual (Table S1 in Appendix S1). The population‐based variant calling approach identified 17 million SNPs and 1.2 million insertions/deletions (indels) (Table S2). Variants were distributed with an average density of 20 SNPs/100‐kb, ranging from 0 to 82. The average transition to transversion (Ts/Tv) ratio was 2.58 (Table S2), which is in line with previous findings in commercial chicken populations (Derks et al., 2018). Samples origin was validated with the principal component analysis (Figure S1).

### 3.1 Population history is responsible for the current autozygosity landscape

**Figure 2:**
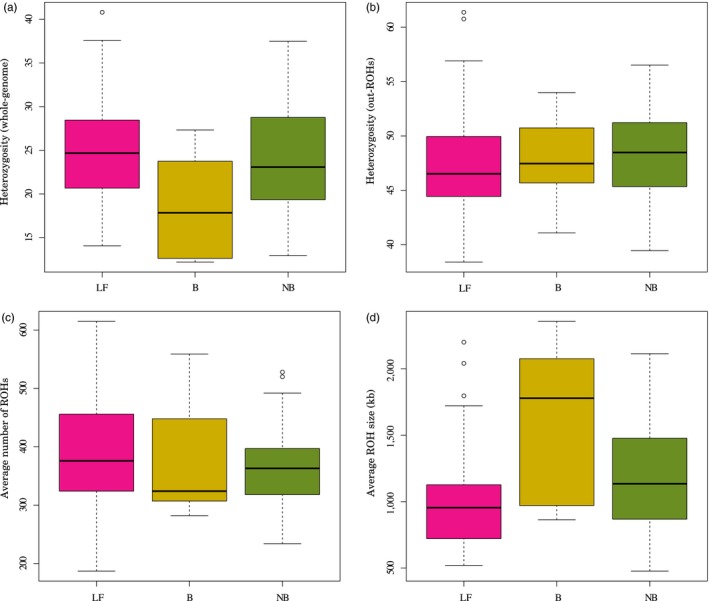
Heterozygosity and runs of homozygosity. (a) Average heterozygosity including ROHs. (b) Average heterozygosity outside ROHs. (c) Average number of ROHs along the genome. (d) Average ROH size in kb. Abbreviations: LF, large fowls (*n* = 49); (b) true bantams (*n* = 7); NB, neo‐bantams (*n* = 39)

**Figure 3:**
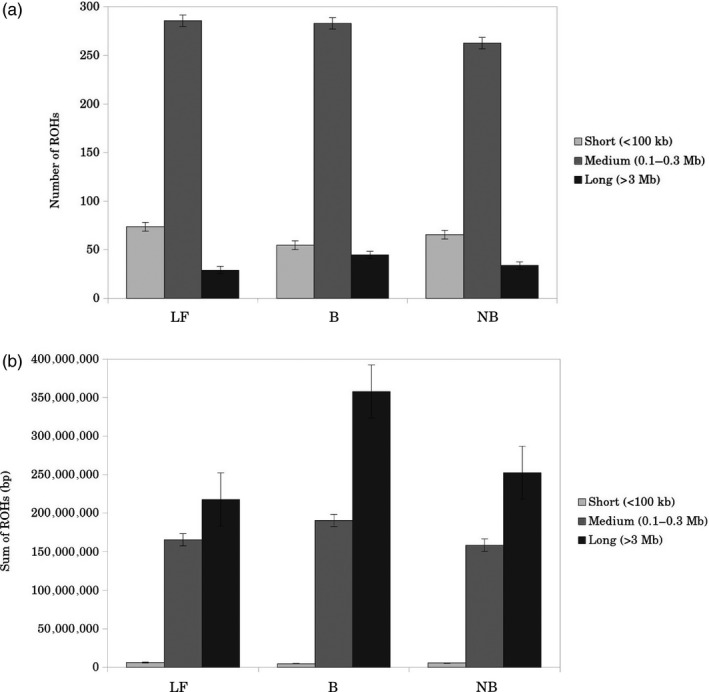
Runs of homozygosity. (a) Average number of short (<100 kb), medium (0.1–3 Mb) and long (>3 Mb) ROHs. (b) Average ROHs size for the three ROH size classes (short, medium and long). Abbreviations: LF, large fowls (*n* = 49); (b) true bantams (*n* = 7); NB, neo‐bantams (*n* = 39)

Genome‐wide autosomal heterozygosity and ROHs were used to investigate the extent and nature of genetic variation in our populations. Whole‐genome heterozygosity ranged from 12.2 to 40.8 SNPs/10‐kb (Table S2 in Appendix S1). On average, neo‐bantams showed slightly lower heterozygosity than their original large fowl counterparts, though the level of heterozygosity was considerably higher than that observed in the bantam breeds (Figure 2a). The level of heterozygosity increased almost two‐fold in all breeds when excluding ROHs, with neo‐bantams showing slightly higher heterozygosity than both source populations (large fowls and bantams) (Table S2 in Appendix S1; Figure 2b). The lower genome‐wide heterozygosity observed in neo‐bantam and bantam breeds is therefore explained by their higher average ROH size (Figure 2d). In fact, the genome of neo‐bantams and bantams is mostly covered by (few) long ROHs (>3 Mb) rather than by small (<100 kb) and medium ROHs (0.1–3 Mb) (Figure 3) (Table S2 in Appendix S1). On average, 25% of the genome in neo‐bantams was covered by long ROHs, 0.6% by short, and 17% by medium ROHs (Table S3 in Appendix S1). Of the bantam breeds, the Eikenburger bantam was the most inbred, with up to 70% of the genome covered by ROHs (Table S3 in Appendix S1).

To further investigate how historical demographic changes have shaped the genomic patterns of homozygosity observed in our populations, we decided to infer past effective population size (*N_e_*). According to our results, the chicken ancestral population size remained stable up to approximately 10,000 years, after which it dropped from an initial *N_e_* of 10^6^ to 10^4^–10^3^ (Figure S3). Both management groups showed a constant flattening population size which has hardly recovered since the bottleneck.

### 3.2 More rare than fixed deleterious variants

**Figure 4:**
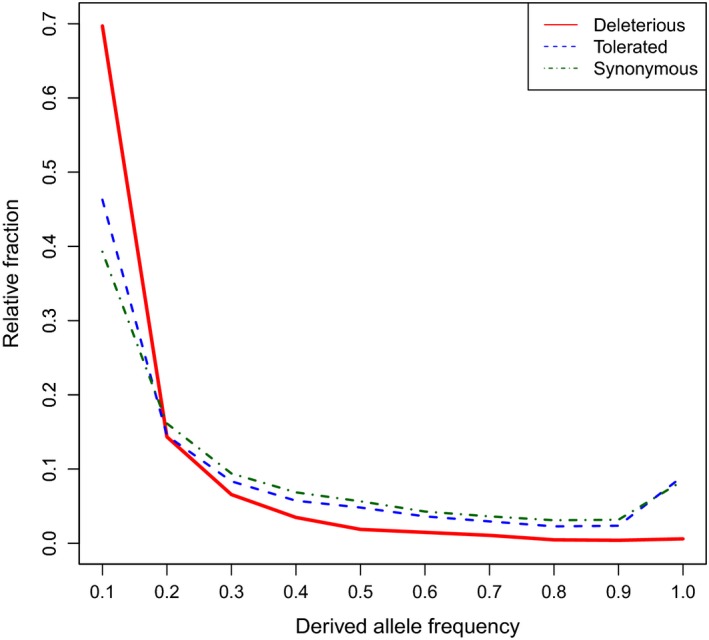
Derived allele frequency spectra of the 39 traditional chicken breeds. Derived allele frequency was inferred for synonymous, nonsynonymous tolerated (SIFT score ≥0.05) and nonsynonymous deleterious (SIFT score <0.05) mutations

The role of genetic drift and purifying selection was investigated by annotating variants with respect to their effects on the amino acid sequence (Table S3). We also annotated alleles as ancestral and derived using three wild *Gallus* species as an outgroup. After filtering for RNA‐seq coverage and 1:1 orthologues, the final set of variants comprised 61,567 synonymous, 16,840 nonsynonymous tolerated, 3,833 nonsynonymous deleterious and 755 loss of function (LoF) mutations. Of the initial set of deleterious variants, 1,674 were classified as deleterious by both SIFT and GERP++. The efficacy of selection at removing deleterious variants from a population was investigated by looking at the distribution of the derived allele frequency (DAF) spectrum. The frequency spectrum showed more rare (DAF <0.1) derived alleles than nearly fixed or fixed deleterious alleles (DAF ≥0.9; Figure 4). We observed similar DAF spectra for large fowls (Figure S4a) and neo‐bantams (Figure S4b), with neo‐bantams showing slightly higher derived allele frequency than large fowl counterparts, even up to a DAF of 0.5. Moreover, neo‐bantams showed fewer nearly fixed or fixed deleterious variants than large fowl counterparts.

### 3.3 The effects of genetic drift on deleterious variation

**Figure 5:**
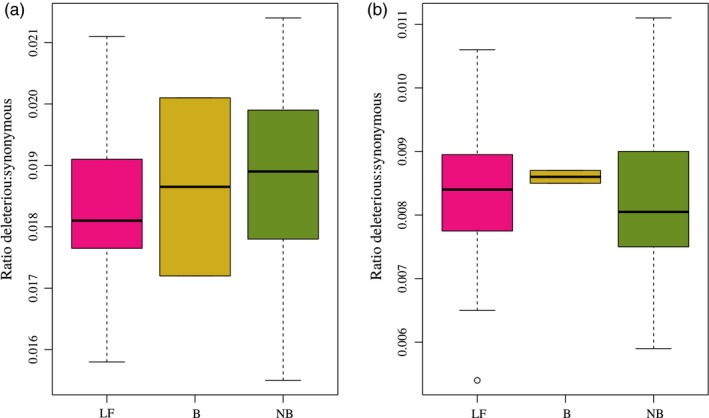
Genetic load expressed as deleterious to synonymous ratio. (a) Genetic load for heterozygous sites. (b) Genetic load for homozygous derived sites. Abbreviations: LF, large fowls (*n* = 35); (b) true bantams (*n* = 3); NB, neo‐bantams (*n* = 22)

In traditional fancy breeds, the total number of derived alleles (heterozygous and homozygous derived) was lower for deleterious and LoF variants relative to putatively neutral ones (synonymous and nonsynonymous tolerated) (Figure S5). Compared with large fowls, neo‐bantams were slightly more enriched in the total number of deleterious and LoF homozygous derived mutations (Figure S5d)**.** Despite these differences in the total number of homozygous derived genotypes, we decided to perform a likelihood ratio test (LRT) to formally test for individual differences between neo‐bantams and large fowls. According to the likelihood ratio test, the number of homozygous derived genotypes was not significantly different between large fowl and neo‐bantam counterparts for deleterious (*p‐value*: 0.730) and LoF (*p‐value*: 0.272) variants (Table 1). Significant were the differences for synonymous (*p‐value*: 3.442e‐08) and nonsynonymous tolerated (*p‐value*: 0.015) variants. We also investigated in each of the three management groups the total genetic burden resulting from the accumulation of deleterious mutations (genetic load; Figure 5). The deleterious to synonymous ratio of heterozygous variants was, on average, lower in large fowls compared with bantam and neo‐bantams. The same ratio when considering homozygous derived variants was, on average, slightly higher in large fowls than neo‐bantams, which, on the other hand, showed extensive variation (Figure 5). Contrary, bantam breeds showed little variation with an average higher deleterious to synonymous ratio than that of large fowls and neo‐bantams.

**Table 1:** Test for elevated homozygosity of derived genotypes per individual between large fowls and neo‐bantams

Functional category	Null model^a^		Alternative model^b^		Likelihood ratio test (LRT)^c^
	MLE	Log‐likelihood	MLE	Log‐likelihood	
Synonymous	p_lf_ = p_nb_ = 0.230	−2,903.70	p_lf_ = 0.229 p_nb_ = 0.232	−2,888.48	Λ = 30.440 *p*‐value: 3.44e−08
Nonsynonymous tolerated	p_lf_ = p_nb_ = 0.209	−902.61	p_lf_ = 0.208 p_nb_ = 0.210	−899.69	Λ = 5.843 *p*‐value: 0.015
Nonsynonymous deleterious	p_lf_ = p_nb_ = 0.070	−323.85	p_lf_ = 0.070 p_nb_ = 0.070	−323.79	Λ = 0.118 *p*‐value: 0.730
Loss of function (LoF)	p_lf_ = p_nb_ = 0.169	−229.89	p_lf_ = 0.167 p_nb_ = 0.171	−229.28	Λ = 1.202 *p*‐value: 0.272

a The null model states that the proportion of homozygous derived genotypes that a large fowl carries is equal to that of the same genotypes carried by a neo‐bantam, so that p_lf_ = p_nb._

b Contrary to the null model, in the alternative model the proportion of homozygous derived genotypes carried by a large fowl individual is different from that of a neo‐bantam (p_lf_ ≠ p_nb_).

c Likelihood ratio test for differences in the number of homozygous derived genotypes per individual between large fowl and neo‐bantams. The chi‐square distribution with one degree of freedom of Λ was used to calculate *p*‐values.

### 3.4 Deleterious variation and demographic history

**Figure 6:**
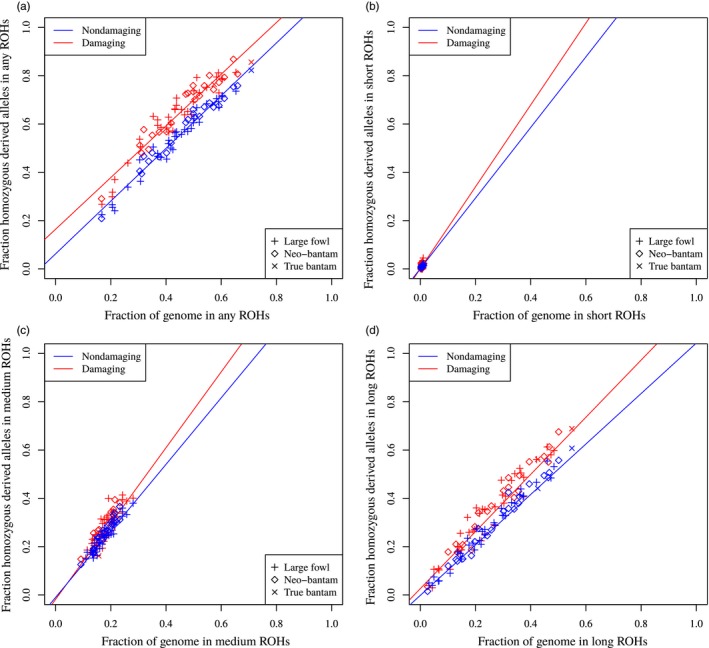
Fraction of the genome covered by ROHs versus the fraction of damaging and nondamaging sites. (a) Any ROH. (b) Short ROHs. (c) Medium ROHs. (d) Long ROHs. Damaging homozygotes are shown for each individual belonging to the three management group, which are identified by different shapes

The study of ROHs offers a new basis for assessing the mechanisms by which demography and selection produce patterns of deleterious variation. The total number of putatively neutral homozygous derived sites was higher than that of damaging sites (Figure S6). Moreover, with the increasing proportion of the genome covered by longer ROHs, the number of homozygous derived variants within ROHs increased for both damaging (*Pearson's correlation*: 0.844, *p‐value*: <2.2e‐16) and nondamaging sites (*Pearson's correlation*: 0.844, *p‐value*: <2.2e‐16). As expected, the number of homozygotes occurring outside ROHs decreased with the fraction of the genome in any ROH, as the genome simply contains fewer ROH‐free regions. A negative correlation between homozygous sites and genome not covered by ROHs confirmed our expectations for damaging (*Pearson's correlation*: −0.624 *p‐value*: 1.261e‐07) (Figure S6b) and nondamaging sites (*Pearson's correlation*: −0.644, *p‐value*: 3.524e‐08; Figure S6a). The fraction of damaging and nondamaging homozygous derived genotypes in ROHs positively correlates with the total genomic ROH coverage (*Pearson correlation*: 0.956, *p‐value*: <2.2e‐16 for damaging; *Pearson correlation*: 0.981, *p‐value*: <2.2e‐16 for nondamaging; Figure 6). We also observed that each ROH size class (short, medium, long and any) is more enriched for deleterious homozygous derived variants than for nondamaging homozygotes. However, this excess in deleterious mutations is particularly evident for any (Figure 6a) and long ROHs (*Pearson correlation*: 0.972, *p‐value*: <2.2e‐16 for damaging; *Pearson correlation*: 0.987, *p‐value*: <2.2e‐16 for nondamaging) (Figure 6d).

## 4 DISCUSSION

In this study, we used whole‐genome sequencing data from traditional fancy breeds of chicken to investigate the consequences of population bottlenecks on genome‐wide patterns of deleterious variation in small populations. To do so, we combined individuals from multiple breeds with similar demographic history into one population to better estimate genetic and deleterious variation. Such approach was most suitable given the low number of individuals per breed (between 1 and 4), which is the direct consequence of the threatened population size of most of these breeds (http://edepot.wur.nl/424249). Even though (slightly) different breeds were grouped into the same population, we expect potential bias to be minimal, as already shown in Bortoluzzi et al. (2018) when using genome‐wide SNP chip data.

In line with the small‐population paradigm, we showed that the size of a population (*N_e_*) is an important evolutionary factor in determining the level of genetic variability and the effectiveness of purifying selection at removing harmful mutations (Caughley, 1994; Charlesworth, 2009). In fact, as we showed, populations of small *N_e_* have a lower genetic diversity and high level of inbreeding, along with being more affected by genetic drift.

In traditional large fowls, the accumulation of deleterious alleles is characteristic of a population that since the domestication bottleneck has persisted for a long period of time at small size. Therefore, in these populations deleterious mutations of especially small effects are expected to have risen in frequency and become fixed (Hedrick, 2001). As several studies have shown, domestication substantially decreases the effective population size and efficacy of purifying selection, which in turn reduces the genetic diversity and increases the mutational load (Moyers et al., 2017). These major effects have been observed in many species despite the multiple domestication centres and large population size of the ancestor (in the case of chicken, the red jungle fowl) (Kanginakudru, Metta, Jakati, & Nagaraju, 2008; Makino et al., 2018; Miao et al., 2013). For example, (Mardsen et al., 2016) recently observed that the dog genome harbours more amino acid changing variants than that of the wild wolf ancestor. The higher proportion of deleterious variants has also led to an increase in the additive genetic load in many dog breeds, which clearly indicates that the efficacy of purifying selection is lowered by strong population contractions accompanying domestication (Cruz, Vila, & Webster, 2008; Mardsen et al., 2016). Similar conclusions have been reached in other domestic animal species (Makino et al., 2018; Schubert et al., 2014) and plants (Liu et al., 2017; Lu et al., 2006). Even though demographic contractions associated with domestication have a major impact on the genome‐wide genetic and deleterious variation, processes that co‐occurred during domestication have recently questioned the role of domestication itself in increasing the mutation load. For example, the shift in mating system from outcrossing to predominantly selfing rice has been suggested to have substantially influenced the occurrence of deleterious mutation in domesticated rice (Liu et al., 2017). It is, however, not to exclude that also the long period of weak and varying artificial selection for desirable traits following domestication could have further reduced *N_e_* (Moyers et al., 2017). Despite the small population size and high genetic load, large fowl breeds retain substantial genetic variation, mainly because of crossing with other breeds performed in the past to maintain a viable population size and nowadays for phenotypic selection (Bortoluzzi et al., 2018). The favourable consequences of genetic exchange (gene flow) observed in our large fowl populations find support in wild species affected by similar drastic population bottlenecks. For example, in the case of the Iberian lynx, the promotion of admixture with Eurasian populations has resulted in less inbred and more genetically diverse populations, potentially more adapted to environmental changes (Abascal et al., 2016).

Contrary to their large fowl counterparts, the extent and nature of deleterious variation in neo‐bantams are characteristics of a population that went through a more recent and severe population decline. In the case of neo‐bantams, the bottleneck is associated with their creation in the last decades (Bortoluzzi et al., 2018). Because of the very small number of founder individuals, neo‐bantams may not yet have had the time to adapt to stochastic demographic and genetic events. This is mostly because the small *N_e_* of neo‐bantams is not large enough to discount the effects of genetic drift. As a result, weakly deleterious mutations accumulate in the genome due to genetic drift, as purifying selection does not have the time to purge these harmful mutations (Luikart, Allendorf, Cornuet, & Sherwin, 1998). The central role of genetic drift observed in our recently bottlenecked populations on deleterious variation is also observed in small populations under natural selection (Abascal et al., 2016; Hedrick, Kardos, Peterson, & Vucetich, 2016; Pollinger et al., 2010; Robinson et al., 2016). In their study, Robinson et al. (2016) showed that a severe bottleneck occurred ~30 generations ago has substantially reduced the already small effective size of the island fox from originally 64 individuals to fewer than a dozen. This recent population contraction has severely affected not only the genetic variation of the species, but also the genetic load. As a result, all island populations show more homozygous deleterious mutations relative to the heterozygous, which, as the authors suggest, have become homozygous likely through strong genetic drift (Robinson et al., 2016).

Although we have shown that the demographic history accompanying the bottleneck and genetic drift are important factors in shaping deleterious variation, inbreeding and artificial selection can also affect the mutational load by increasing the probability of harmful mutations to become homozygous. In fact, in homozygous state, these recessive deleterious mutations can potentially lower an individual fitness (inbreeding depression) (Bosse et al., 2019). Small populations are more prone to suffer from inbreeding depression, as the probability of mating between relatives is high. To test whether the high level of inbreeding observed in our populations affects the deleterious variation landscape, we looked at the distribution of deleterious mutations inside and outside ROHs following Szpiech et al. (2013). In line with results in humans (Szpiech et al., 2013), domesticated pig (Bosse et al., 2019), commercial chicken (Bosse et al., 2019) and cattle (Zhang, Guldbrandtsen, Bosse, Lund, & Sahana, 2015), we found ROHs to be proportionally more enriched in homozygous deleterious alleles than the rest of the genome. However, when looking at the ROH size classes, long ROHs, which are an indication of recent inbreeding, were significantly more enriched than any other size class. This pattern was particularly clear in neo‐bantams, which supports the role of both inbreeding and genetic drift in increasing the occurrence of deleterious mutations in homozygous state.

In a recent study on commercial chicken lines, Derks et al. (2018) observed that putative highly deleterious variants can be rare in populations of small effective size if specific breeding programmes aiming to select individuals against inbreeding depression are in place. Therefore, the presence of a breeding programme counterbalances the effects of inbreeding and strong artificial selection. In traditional breeds, as well as in small populations under natural selection, the risk of increasing an individual mutational load is considerably higher, because breeding and conservation programmes are often not in place to genetically manage these populations (Bortoluzzi et al., 2018). Moreover, as mating between family members is intentionally pursued to select for specific traits, the proportion of homozygous segments in individual genomes is expected to substantially increase along with that of slightly deleterious mutations. Therefore, we expect the viability of the traditional breeds investigated in this study to strongly depend on future breeding preferences, which, if not genetically managed, are likely to limit the full exploitation of their genetic potential.

## 5 CONCLUSIONS

In this study, we showed that the timing and nature of a population bottleneck can substantially shape the deleterious variation landscape in small populations. In particular, we showed that populations kept at small size for long period of time since the bottleneck have a reduced burden of deleterious alleles compared with recently bottlenecked populations. The reduced deleterious burden in these populations, which is also linked to a reduced number and total length of ROHs across the genome, is likely responsible for their genetic success.

According to our study, facilitating purging of deleterious mutations through inbreeding avoidance should be at the core of future breeding and conservation programmes in small populations (de Cara, Villanueva, Toro, & Fernandez, 2013). However, genomic information on deleterious variation can, and should, be incorporated and used in the development of conservation programmes that assure the long‐term survival and enhance the genetic diversity of small populations. Fitness‐related traits should also be considered to better measure potential fitness consequences at the individual and population level associated with recessive deleterious mutations.

## Data Availability

Whole‐genome sequencing data from this study have been submitted to the European Nucleotide Archive (ENA) under accession number PRJEB34245. Source codes for running the ROH pipeline have been deposited in https://github.com/cbortoluzzi/ROHs.
