# Supplementary material for: The type of bottleneck matters: Insights into the deleterious variation landscape of small managed populations
Source: Evol Appl. 2019 Sep 30;13(2):330–41. doi: 10.1111/eva.12872 (PMC6976952; doi:10.1111/eva.12872)

Additional file 2

List of Figures

S1 **Principal Component Analysis (PCA)** 6
S2 **Heterozygosity along chromosome 1** 7
S3 **Estimates of past effective population size** 8
S4 **Derived allele frequency of large fowl and neo-bantam breeds** 9
S5 **Distribution of the number of heterozygous and homozygous genotypes per individual** 10
S6 **Number of nondamaging and damaging homozygous derived sites inside and outside ROHs** . . 11

List of Tables

S1 **Overview of sequenced samples included in the study** 2
S2 **Summary of variants and indels kept after variant calling and post-filtering** 3
S3 **Functional annotation of variants** 4
S4 **SIFT classification** 4
S5 **Overview of samples used in SMC++** 5

Table S1: Overview of sequenced samples included in the study

Abbreviations: NL: the Netherlands; LF: Large fowl; NB: neo-bantam; B: true bantam

Breed name	No. individual	Management	Origin
Assendelft Fowl	3	LF	NL
Assendelft Fowl Bantam	2	NB	NL
Barnevelder	4	LF	NL
Barnevelder Bantam	3	NB	NL
Brabanter	3	LF	NL
Brabanter Bantam	3	NB	NL
Breda Fowl	3	LF	NL
Breda Fowl Bantam	3	NB	NL
Chaam Fowl	2	LF	NL
Creeper	2	LF	NL
Drenthe Fowl	1	LF	NL
Drenthe Fowl Bantam	1	NB	NL
Drenthe Fowl Bolstaat	2	LF	NL
Dutch Bantam	3	B	NL
Dutch Booted Bantam	1	B	NL
Dutch Fowl	4	LF	NL
Dutch Fowl Bantam	2	NB	NL
Dutch Owl Bearded	3	LF	NL
Dutch Owl Bearded Bantam	3	NB	NL
Dutch Polish Bearded	2	LF	NL
Dutch Polish Bearded Bantam	2	NB	NL
Dutch Polish non Bearded	2	LF	NL
Dutch Polish non Bearded Bantam	2	NB	NL
Eikenburger Bantam	2	B	NL
Frisian Fowl	4	LF	NL
Frisian Fowl Bantam	2	NB	NL
Gallugs sonneratii	1	LF	?
Gallus gallus	1	LF	?
Gallus lafayetii	1	LF	?
Gallus varius	1	LF	?
Groninger Mew	4	LF	NL
Groninger Mew Bantam	4	NB	NL
Java	1	B	NL
Kraienkoppe	3	LF	NL
Kraienkoppe Fowl Bantam	3	NB	NL
Lakenvelder	3	LF	NL
Lakenvelder Bantam	3	NB	NL
North Holland Blue	1	LF	NL
North Holland Blue Bantam	1	NB	NL
Schijndelaar	1	LF	NL
Schijndelaar Bantam	1	NB	NL
Welsummer	4	LF	NL
Welsummer bantam	4	NB	NL

Table S2: Summary of variants and indels kept after variant calling and post-filtering
The fifth column shows the average number of variants in bins of 10 Kb, the sixth column the total number of transitions, the seventh the total number of transversion, and the last column the average transition to transvertion ratio

Chrom	# records	# SNPs	# Indels	Var/10 Kb	Ts	Tv	Ts/Tv
1	3877122	3551646	257622	19.76	2445981	1066838	2.29
2	2956232	2704405	200314	19.76	1862453	813223	2.29
3	2198459	2006472	153563	19.75	1390239	593274	2.34
4	1788716	1628925	128255	19.59	1130606	478874	2.36
5	1188298	1082810	84449	19.86	758333	311177	2.44
6	793498	728334	51273	22.69	504249	216418	2.33
7	748910	684870	51251	20.27	481039	196259	2.45
8	562037	514773	38119	18.75	364158	144468	2.52
9	513764	471603	33553	21.77	333464	132935	2.51
10	413767	379651	27350	20.75	271128	104380	2.60
11	359527	328316	25389	17.78	233449	91080	2.56
12	425025	389956	28005	21.31	278949	106399	2.62
13	369740	340674	22678	20.65	242232	94321	2.57
14	317877	291637	20488	20.37	210130	77715	2.70
15	238505	219014	15522	18.67	158287	57784	2.74
16	13984	13067	547	21.18	8661	4225	2.05
17	211597	195634	12466	20.22	142572	50581	2.82
18	224726	207088	13551	20.31	149000	55790	2.67
19	192302	177608	11523	19.26	129562	46006	2.82
20	281958	260172	17095	19.99	187485	69555	2.70
21	140593	129769	8432	20.49	94308	33809	2.79
22	67960	62277	4300	14.36	44396	16811	2.64
23	122947	113664	7112	21.23	83003	28835	2.88
24	133926	124455	7314	21.32	91619	31403	2.92
25	51268	46927	3196	17.61	33596	12340	2.72
26	115278	106660	6569	21.66	78615	26572	2.96
27	125699	115853	6943	22.24	79455	34491	2.30
28	107433	98251	6999	21.57	71153	25427	2.80
30	316	247	57	10.53	167	73	2.29
31	718	687	7	14.36	407	272	1.50
32	1327	1164	126	16.58	889	243	3.66
33	32602	29107	2735	19.75	20883	7532	2.77
**Total**	**18576111**	**17005716**	**1246803**	**19.96**	**11880468**	**4929110**	**2.58**

Table S3: Functional annotation of variants

The table reports for each functional class the total number of variants with most severe consequences identified in the 59 highly covered samples for which the ancestral and derived state of a variant was inferred

**Consequences of variant**	**Total number**
Splice donor	846
Splice acceptor	658
Stop gained	536
Frameshift	1469
Stop lost	123
Start lost	270
Inframe insertion	528
Inframe deletion	558
Protein altering variant	41
Missense variant	66599
Splice region	33569
Synonymous variant	154855
Stop retained	77
Mature miRNA	266
5'UTR	24091
3'UTR	128003
Intron variant	8831024
Non coding transcript	1
Upstream gene	1269188
Downstream gene	927319
Intergenic	7800108
**Sum (LoF)**	4988
**Sum(protein-coding)**	226442
**Sum (all)**	19240129

Table S4: SIFT classification

The table reports the total number of missense variants classified by SIFT as deleterious and tolerated

**SIFT**	**Total number**
deleterious - low confidence	4519
tolerated - low confidence	10205
deleterious	12266
tolerated	61202

Table S5: Overview of samples used in SMC++

The table reports the individuals used to infer past effective population size and demographic history. Management is the group the sample belongs to and coverage is the average genome-wide coverage calculated after mapping. The percentage of missing sites was calculated on the filtered vcf files. Abbreviations: LF, large fowl; NB, neo-bantam; B, true bantam

Sample ID	Breed name	Management	Coverage	Fraction missing sites
ADIV00007	Groninger Mew	LF	15.398	4.175
ADIV00010	Groninger Mew	LF	12.451	6.634
ADIV00022	Groninger Mew	LF	15.962	4.125
ADIV00025	Groninger Mew Bantam	NB	14.655	4.237
ADIV00031	Groninger Mew Bantam	NB	16.425	4.091
ADIV00040	Groninger Mew Bantam	NB	14.613	4.251
ADIV00048	Lakenvelder	LF	15.699	4.113
ADIV00050	Lakenvelder	LF	14.139	4.254
ADIV00052	Lakenvelder	LF	15.685	4.150
ADIV00055	Lakenvelder Bantam	NB	14.032	4.213
ADIV00056	Lakenvelder Bantam	NB	16.571	4.035
ADIV00058	Lakenvelder Bantam	NB	18.061	4.031
ADIV00095	Frisian Fowl	LF	17.250	4.017
ADIV00097	Frisian Fowl	LF	16.211	4.109
ADIV00100	Frisian Fowl	LF	15.770	4.071
ADIV00127	Dutch Fowl Bantam	NB	15.415	4.200
ADIV00128	Dutch Fowl Bantam	NB	18.426	4.051
ADIV00130	Dutch Polish Bearded	LF	15.802	4.067
ADIV00131	Dutch Polish Bearded	LF	15.842	4.129
ADIV00147	Dutch Owl Bearded	LF	17.091	4.040
ADIV00151	Dutch Owl Bearded	LF	17.136	4.030
ADIV00165	Dutch Owl Bearded Bantam	NB	18.361	3.986
ADIV00170	Dutch Owl Bearded Bantam	NB	15.869	5.252
ADIV00189	Dutch Polish non Bearded Bantam	NB	33.530	3.934
ADIV00190	Dutch Polish non Bearded Bantam	NB	16.136	4.051
ADIV00203	Kraienkoppe	LF	17.009	4.048
ADIV00209	Kraienkoppe	LF	16.479	4.079
ADIV00317	Barnevelder	LF	24.184	3.967
ADIV00322	Barnevelder	LF	28.533	3.983
ADIV00334	Barnevelder	LF	16.423	4.155
ADIV00346	Barnevelder Bantam	NB	16.031	4.317
ADIV00348	Barnevelder Bantam	NB	17.455	4.119
ADIV00351	Welsummer	LF	11.247	8.099
ADIV00352	Welsummer	LF	12.222	4.451
ADIV00356	Welsummer	LF	15.606	4.203
ADIV00384	Breda Fowl	LF	16.303	4.128
ADIV00389	Breda Fowl	LF	19.623	4.206
ADIV00406	Chaam Fowl	LF	25.303	3.892
ADIV00408	Chaam Fowl	LF	13.620	4.230

Figure S1: Principal Component Analysis (PCA). The principal component analysis was performed on 95 samples from 39 traditional Dutch chicken breeds. Samples belonging to the same management group are here shown with the same color and shape. Abbreviations: LF, large fowls; NB, neo-bantams; B, true bantams

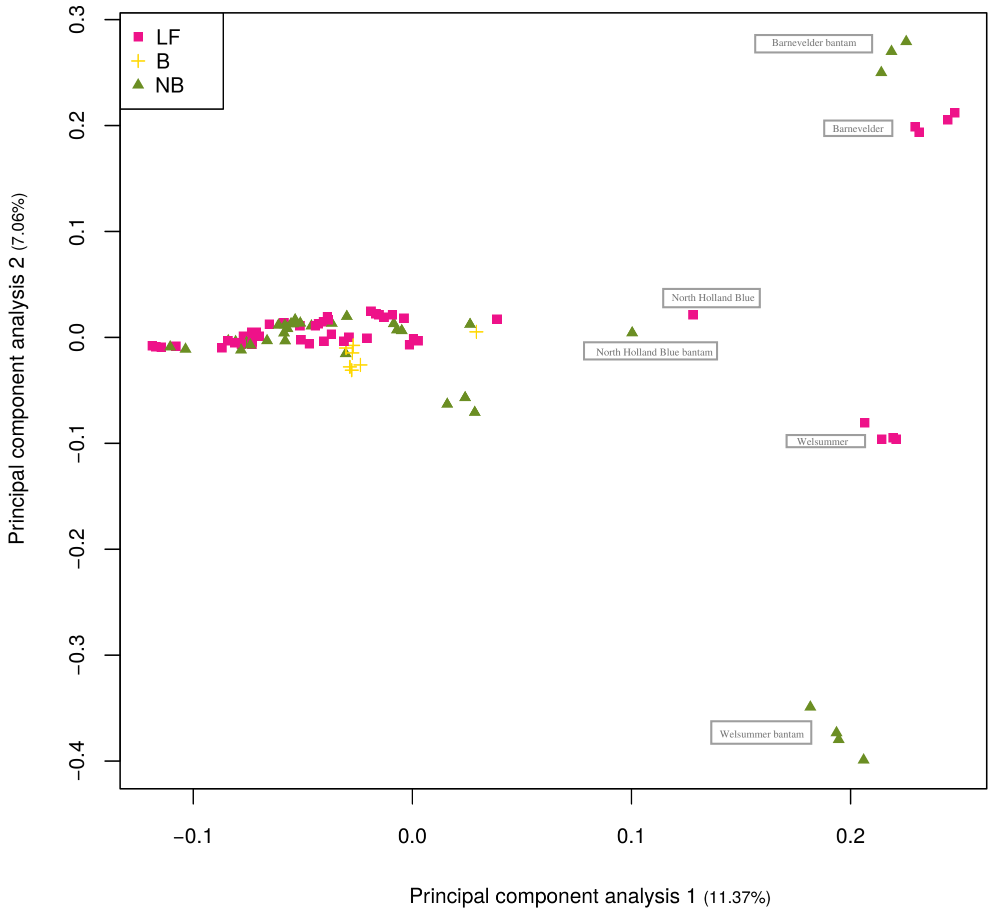

Figure S2: Heterozygosity along chromosome 1. Heterozygosity is shown for **(a)** an Eikenburger bantam and **(b)** a Drenthe fowl bolstaat individual. The x-axis shows the position in base pairs (bps) along chromosome 1 and the y-axis displays the corrected number of heterozygous SNPs counted in non-overlapping windows of 10 Kb

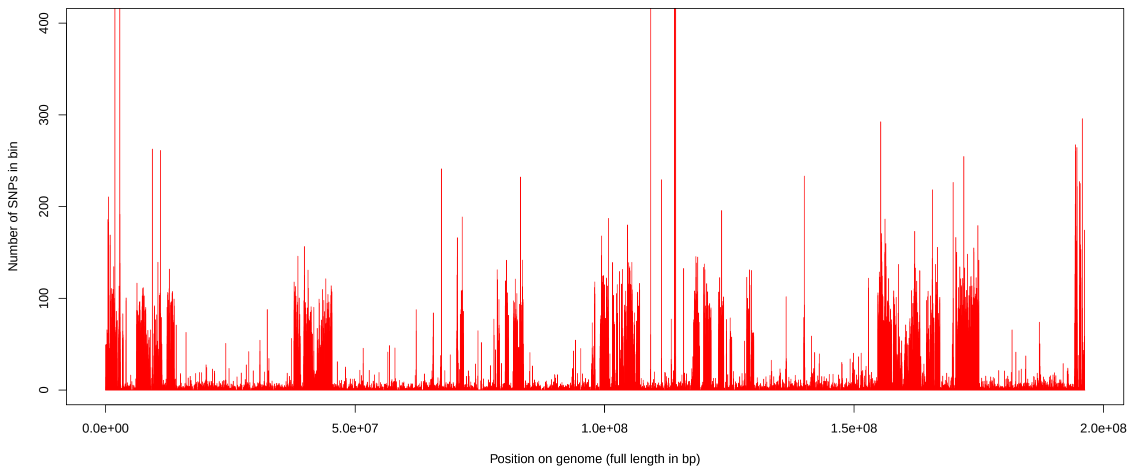

(a)

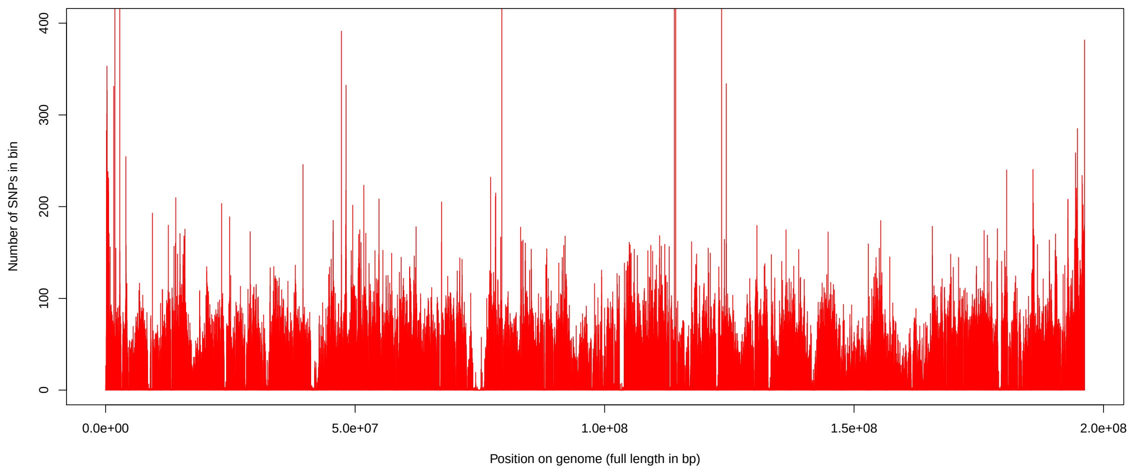

(b)

Figure S3: Estimates of past effective population size. Changes in effective population size are shown for (**A**) large fowl and (**B**) neo-bantam breeds. The x-axis shows the years back in time and the y-axis the estimated effective population size, N_e. Abbreviations: Barnev, Barnevelder; BarnevB, Barnevelder bantam; BreFw, Breda fowl; ChamFw, Chaam fowl; DFwB, Dutch fowl bantam; DOwBd, Dutch owl bearded; DOwBdB, Dutch owl bearded bantam; DPBd, Dutch Polish bearded; DPnBdB, Dutch Polish non bearded bantam; FriFw, Frisian fowl; GrMw, Groninger mew; GrMwB, Groninger mew bantam; KraiK, Kraienkoppe; LakVe, Lakenvelder; LakVeB, Lakenvelder bantam; Welsum, Welsummer; WelsumB, Welsummer bantam

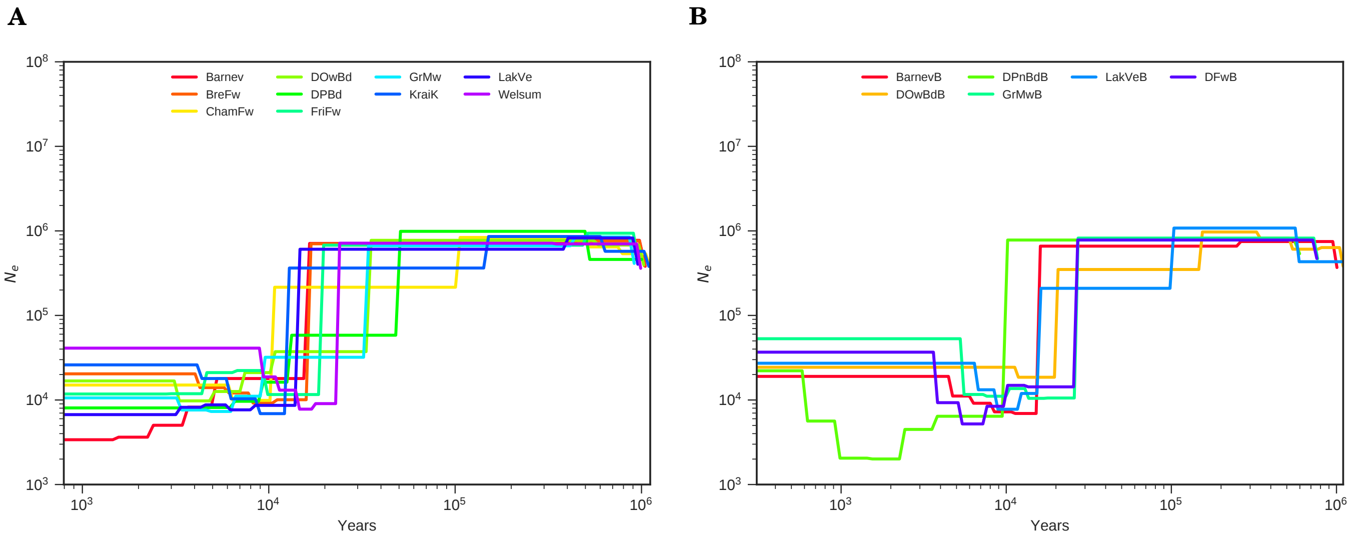

Figure S4: Derived allele frequency of large fowl and neo-bantam breeds. **A.** Derived allele frequency of large fowl breeds (n=35). **B.** Derived allele frequency of neo-bantam breeds (n=22). Derived allele frequency was inferred for synonymous, missense deleterious (SIFT < 0.05) and missense tolerated (SIFT ≥ 0.05) mutations

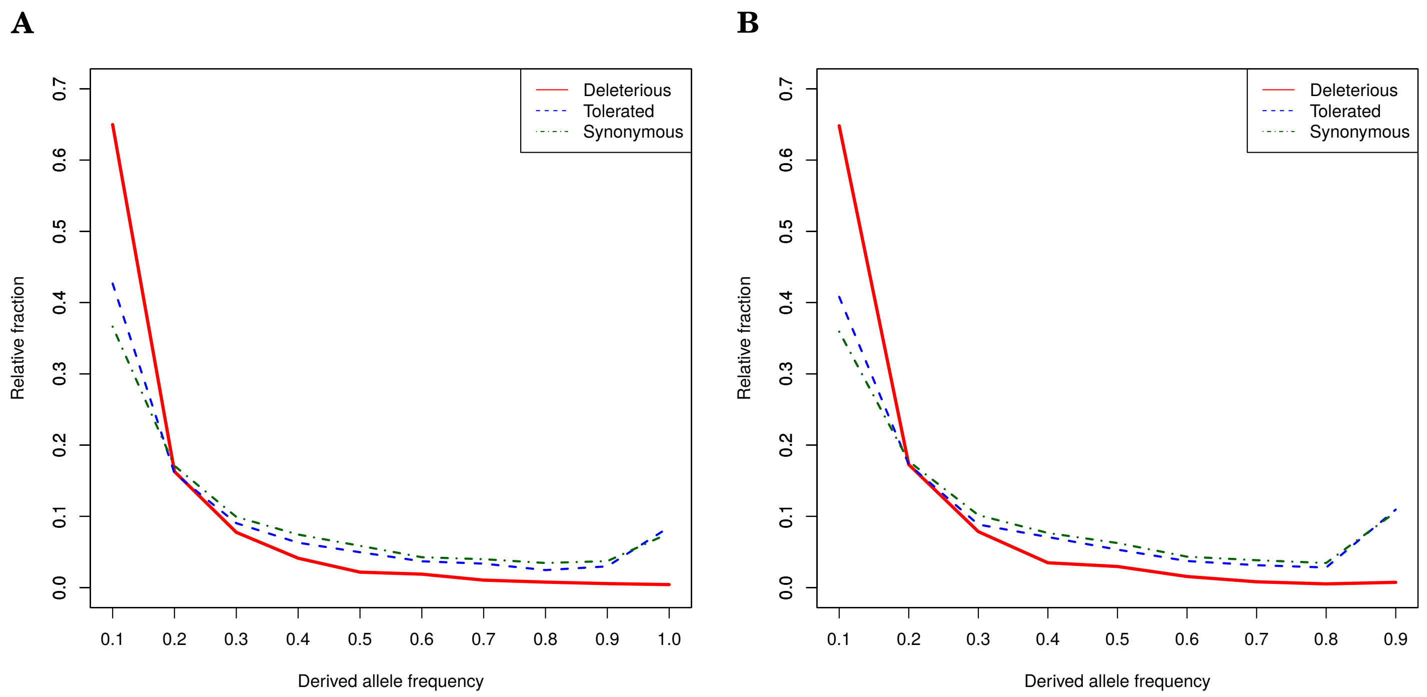

Figure S5: Distribution of the number of heterozygous and homozygous genotypes per individual. **A.** Total number of putatively neutral heterozygous genotypes. **B.** Total number of putatively neutral homozygous derived genotypes. **C.** Total number of putatively deleterious heterozygous genotypes. **D.** Total number of putatively deleterious homozygous derived genotypes. Traditional breeds are here grouped based on their management into large fowls (LF), neo-bantam (NB), and true bantam (B)

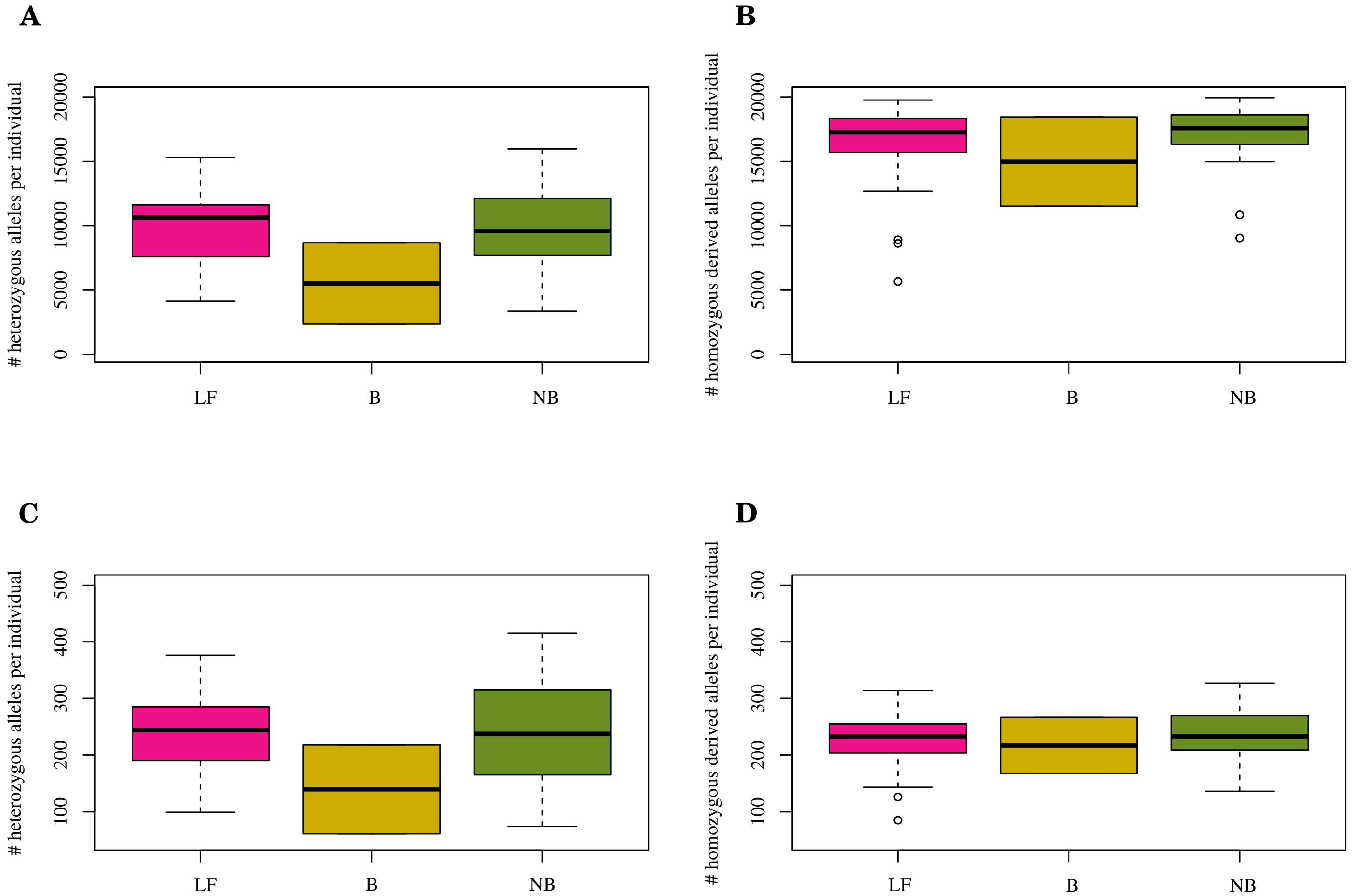

Figure S6: Number of nondamaging and damaging homozygous derived sites inside and outside ROHs. **A.** Number of nondamaging homozygotes. **B.** Number of damaging homozygotes. Green points represent the number of nondamaging (A) and damaging (B) homozygotes outside ROHs

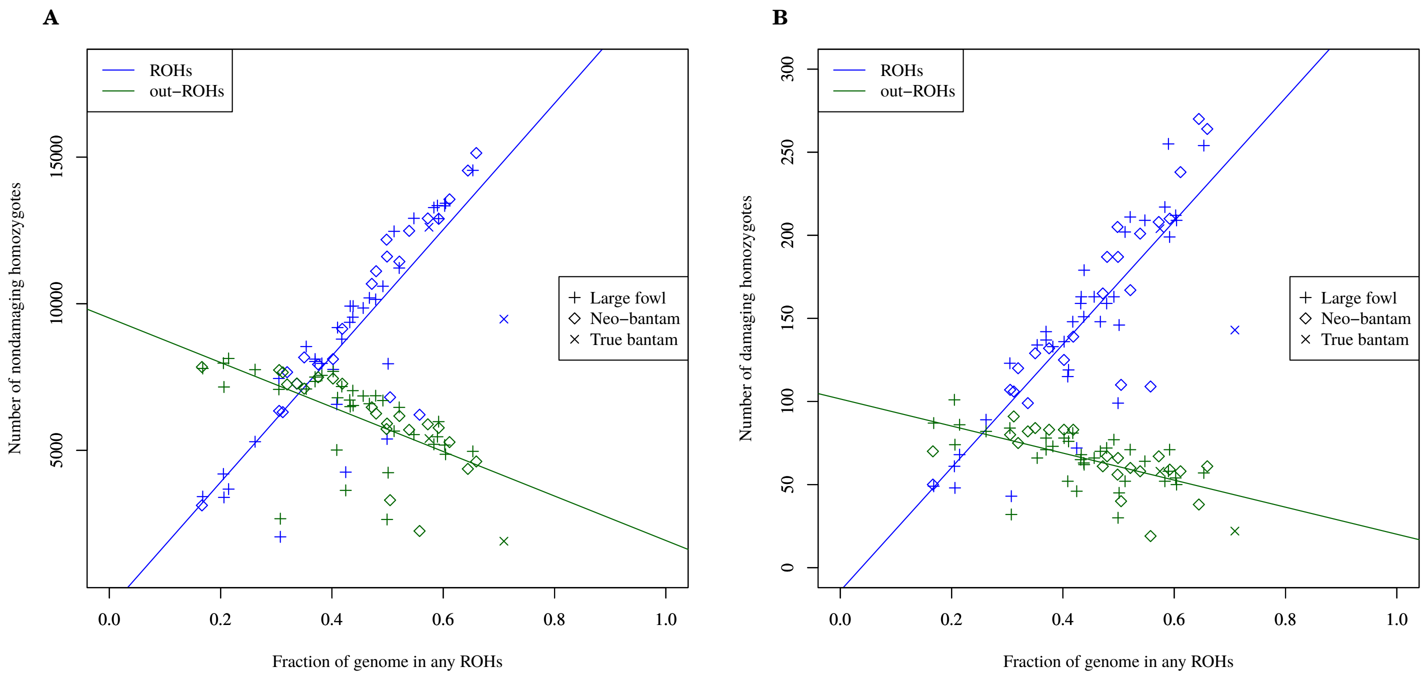

Supplement: Supplementary file 2 [file EVA-13-330-s002.pdf]
