# Supplementary material for: The type of bottleneck matters: Insights into the deleterious variation landscape of small managed populations
Source: Evol Appl. 2019 Sep 30;13(2):330–41. doi: 10.1111/eva.12872 (PMC6976952; doi:10.1111/eva.12872)
Supplement: Supplementary file 3 [file EVA-13-330-s003.docx]

**Supplementary Information**

**S1 Text**

**Alignment and variant calling**

Reads were trimmed using sickle v.1.33 (Joshi & Fass, 2011) before being mapped to the chicken genome (GenBank Accession: GCA_000002315.3) with the Burrows Wheeler Aligner (BWA) v.0.7.15 (Li & Durbin, 2009). Mapping was performed with the default settings. Duplicate reads were removed using the *rmdup* function of samtools v.1.19 (Li et al., 2009). The GATK IndelRealigner was then used to perform realignment of reads around indels (McKenna et al., 2010). Genome-wide coverage and mapping quality were evaluated with Qualimap v.2.2 (García-Alcalde et al., 2012). We performed a population-based variant calling using Freebayes v.0.9.10 (Garrison & Marth, 2012). The following criteria were used: (1) –min-base-quality 10 (a support base quality > 10), (2) –min-mapping-quality 20 (a support mapping quality > 20), (3) –min-alternate-fraction 0.2 (at least 20% of reads supporting the alternative allele), and a –min-alternate-count 2 (at least 2 reads supporting the alternative allele). To reduce the number of missed variants, variants of each individual were filtered out if they did not meet the coverage requirements, which were a minimum read depth of 4X and a maximum of 2.5 times the average individual genome-wide coverage. The false discovery rate was reduced by performing additional post-processing using Bcftools v.1.4.1 (Li et al., 2009), setting (1) a phred quality score > 30, (2) allele count supporting the alternative allele > 2, (3) maximum number of 10 alleles, (4) variants located within 3 bp of an indel, and (5) call rate < 0.70.

**Likelihood ratio test for elevated proportion of homozygosity of derived genotypes**

We tested whether the number of homozygous derived genotypes per individual differed between large fowls and neo-bantams, implementing the approach developed in Robinson et al. (2016). For a given set of genotypes *g*, the number of homozygous derived alleles that an individual carries follows a binomial distribution with parameters *g* and *p*, where *p* is the proportion of SNPs for which an individual is homozygous derived. According to the null model, the proportion of homozygous derived genotypes that a large fowl individual carries is equal to the proportion of the same genotypes carried by a neo-bantam individual, as expressed by the formula *p_lf_* = *p_nb_*_._ Therefore, the null model was:

$l\left( p\vee x,g \right)=\sum_{j=1}^{57} x_{j}log\left( p \right)+\left( g_{j}-x_{i} \right)log\left( 1-p \right)$

where *x_j_* is the number of homozygous derived genotypes for individual *j* and *g_j_* the total number of called genotypes in individual *j*. Since we assumed similar proportion of homozygous derived alleles between neo-bantams and large fowls, the null model was calculated over the 57 individuals (35 large fowl and 22 neo-bantam individuals). Under the alternative model, differences in the number of homozygous derived genotypes were expected between large fowls and neo-bantams, so that *p_lf_* ≠ *p_nb_*:

$\begin{aligned} l\left( p_{lf},p_{nb}\vee x,g \right)=\sum_{j=1}^{35} x_{lf}log\left( p_{lf} \right)+\left( g_{lf}-x_{lf} \right)log\left( 1-p_{lf} \right) \\ \begin{matrix} 1-p_{nb} \\ +\sum_{j=1}^{22} x_{nb}log\left( p_{nb} \right)+\left( g_{nb}-x_{nb} \right)log \end{matrix} \end{aligned}$

where $x_{lf}$ and $x_{nb}$ is the number of homozygous derived genotyped of the large fowls (*n*=35) and neo-bantams (*n*=22), respectively, and *g_lf_* and *g_nb_* the total number of called genotypes in the large fowls and neo-bantams. We then tested whether the alternative model fit the data better than the null model using a likelihood ratio test (LRT) as:

$\Lambda=-2\left( l\left( p\vee x,g \right)-l\left( p_{lf},p_{nb}\vee x,g \right) \right)$

where $\Lambda$ is $\chi^{2}$ distribution with one degree of freedom, from which we calculated p-values. The likelihood ratio test was separately calculated for synonymous, missense tolerated, missense deleterious, and loss-of-function variants.

**References**

García-Alcalde, F., Okonechnikov, K., Carbonell, J., Cruz, L. M., Götz, S., Tarazona, S., ... & Conesa, A. (2012). Qualimap: evaluating next-generation sequencing alignment data. *Bioinformatics*, *28*(20), 2678-2679.

Garrison, E., & Marth, G. (2012). Haplotype-based variant detection from short-read sequencing. *arXiv preprint arXiv:1207.3907*.

Joshi, N. A., & Fass, J. N. (2011). Sickle: A sliding-window, adaptive, quality-based trimming tool for FastQ files (Version 1.33)[Software].

Li, H., & Durbin, R. (2009). Fast and accurate short read alignment with Burrows–Wheeler transform. *bioinformatics*, *25*(14), 1754-1760.

Li, H., Handsaker, B., Wysoker, A., Fennell, T., Ruan, J., Homer, N., ... & Durbin, R. (2009). The sequence alignment/map format and SAMtools. *Bioinformatics*, *25*(16), 2078-2079.

McKenna, A., Hanna, M., Banks, E., Sivachenko, A., Cibulskis, K., Kernytsky, A., ... & DePristo, M. A. (2010). The Genome Analysis Toolkit: a MapReduce framework for analyzing next-generation DNA sequencing data. *Genome research*, *20*(9), 1297-1303.Joshi, N. A., & Fass, J. N. (2011). Sickle: A sliding-window, adaptive, quality-based trimming tool for FastQ files (Version 1.33)[Software].

Robinson, J. A., Ortega-Del Vecchyo, D., Fan, Z., Kim, B. Y., Marsden, C. D., Lohmueller, K. E., & Wayne, R. K. (2016). Genomic flatlining in the endangered island fox. *Current Biology*, *26*(9), 1183-1189.
